# Enhanced vehicle localization with low-cost sensor fusion for urban 3D mapping

**DOI:** 10.1371/journal.pone.0318710

**Published:** 2025-05-02

**Authors:** Sheraz Shamim, Syed Riaz un Nabi Jafri

**Affiliations:** Department of Electronic Engineering, NED University of Engineering and Technology, Karachi, Pakistan; Tongji University, CHINA

## Abstract

This research paper presents the design and development of an indigenous low cost Mobile Mapping System (MMS) for urban surveying applications. The MMS is comprised of economical Hokuyo-30LX 2D laser scanners, vision sensors, Global Positioning System (GPS) and various odometric sensors that can be installed on car like moving platform. The run time sensorial data is interfaced, processed and recorded using Robot Operating System (ROS). The live laser scan is utilized for the pose estimation using Simultaneous Localization and Mapping (SLAM) technique. In absence of valid SLAM estimation and frequent GPS outages, a multimodal sensor fusion framework for the enhanced pose correction has been developed using Kalman Filter (KF) by incorporating the Inertial Measurement Unit (IMU) and wheel odometric data along with SLAM and GPS data. The corrected pose is utilized for the 3D point cloud mapping by incorporating laser scans perceived periodically from various 2D laser scanners mounted on the MMS. The custom-made installation scheme has been followed for mounting three 2D laser scanners at horizontal, vertical and inclined orientations. The efficacy of the developed map has employed for extraction of road edges and associated road assets by establishing the lucrative classification technique of the point cloud using Split and Merge segmentation and Hough transformation. The surveying to map development time has significantly reduced and the mapping results have found quite accurate when matched with the ground truths. Furthermore, the comparison of the developed maps with ground truths and GIS tools reveals the highly acceptable accuracy of the generated results which have found very nearly aligned with the actual urban environment features. In comparison to the existing global MMS variants, the presented MMS is quite affordable solution for limited financial resourced business entities.

## 1. Introduction

The necessity of consistently collecting and updating geospatial data on road networks and associated assets has increased extensively over the last two decades [1]. The collected geospatial data of surveyed urban regions assists in numerous applications, including 3D infrastructure modeling, generation of high-definition (HD) maps, navigation of autonomous vehicles, smart city management and geographic information system (GIS) enhancement [2,3]. A mobile mapping system (MMS) is generally used to collect survey data. The MMS is typically a multisensor integration of proprioceptive and exteroceptive sensing devices that are mounted on a moving platform such as a car [4]. Highly accurate and precise positioning information on surveying vehicles is essentially required for error-prone georeferenced data collection in outdoor environments. The Global Navigation and Satellite System (GNSS) is widely used to accurately determine the location and speed of surveying vehicles. However, the operation can be severely affected by urban canyons, weather conditions and multipath interference, which cause localization and navigational errors to increase over time [5].

To improve localization during GPS outages, additional odometric sensors, such as Inertial Measurement Unit (IMU) and wheel encoders, have been utilized by the research community. To palliate the inherent errors of odometric sensors, various filtering techniques, such as the Kalman Filter (KF), have been adopted by researchers [6]. In addition, various scholars have incorporated cameras, laser scanners and radar to mitigate the localization errors using the distinct environmental features of surveyed vicinities [7]. The robotic research community has introduced the Simultaneous Localization and Mapping (SLAM) technique using the fusion of odometric and laser scan data for estimating vehicle poses and environmental maps [8]. Similarly, the fusion of vision and GNSS information has been carried out in multiple studies to determine the localization of a moving vehicle in real time [9]. A car-based mapping solution has been presented by scholars by integrating distinct sensors along with GNSS data in the KF framework [10]. Another research group has developed the state-of-the-art MMS to process high-end computations for feature extraction and classification of road assets such as electric poles and road markings [11]. In general, the MMS capability for precise localization, mapping and road asset classification requires computationally intensive processing of high-resolution sensorial data with sufficient storage and processing resources [12]. This makes the majority of existing MMS consoles excessively expensive and unaffordable for limited financial resources projects for low-range surveys.

**Fig 1:**
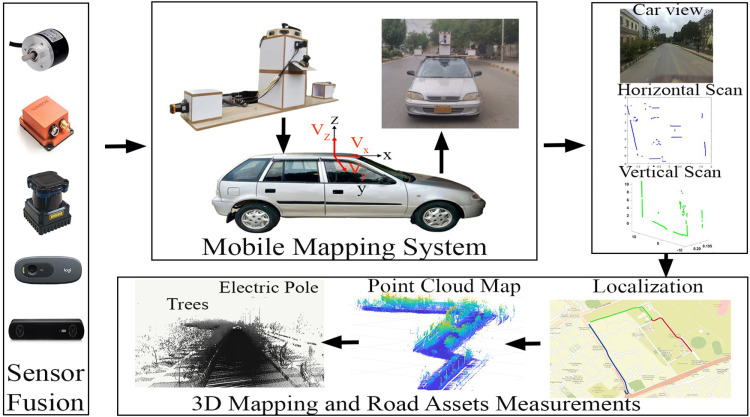
Developed Mobile Mapping System (MMS) with 3D maps and Road asset measurement.

Therefore, this research presents a customized low-cost car-based MMS for the development of georeferenced maps and road asset classification. The developed MMS is equipped with multiple short-range 2D laser scanners, GPS, IMU, cameras and wheel odometers to provide accurate localization and 3D point cloud mapping solutions for surveyed vicinities via KF implementation as shown in Fig 1. In addition, the processing of point cloud maps has accomplished segmentation, road edge extraction and road asset classification using affordable and easy procedures for less-resourced business entities. To show all the contributions systematically, this paper is organized as follows. Section II describes an overview of existing MMS and their related works. Section III elaborates the mechanical and instrumentation design of the developed MMS. Section IV presents the methodology for MMS localization, point cloud mapping and classification for road asset management. Section V explains the actual surveying results of the outdoor environments. Finally, the conclusions of the presented work are discussed.

## 2. Related work

Continuous performance evolution has been occurred since the last century in the MMS field due to advancements in computational and sensing technologies. This section briefly presents the operation of existing vehicle-based MMS modules for surveying and mapping applications of targeted outdoor environments.

The performance of the MMS is highly dependent on the accuracy of the localization of the moving platform at which the MMS was installed. A research group has presented a real-time vehicle localization solution by using low-cost loosely coupled sensors with an Unscented Kalman Filter (UKF) [13]. Scholars have fused inertial and laser scans with RTK measurements via a tightly coupled method to localize vehicles in urban environments [14]. A similar tightly coupled localization approach for urban vicinities was generated by adopting the extended KF in published research [15]. A combination of GNSS, laser scan and odometry data has been incorporated by researchers to estimate the SLAM of rail vehicles [16]. In addition, navigation solutions for moving platforms using GNSS, odometry and laser SLAM have been proposed by many other researchers [17].

**Fig 2:**
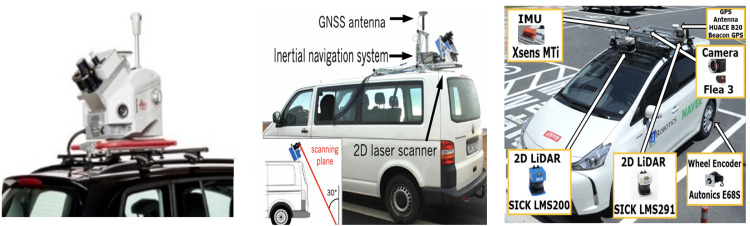
Car-based mobile mapping platforms (left) Leica Pegasus (middle) Developed at the University of Bonn (right) Developed at KAIST.

The MMS installed on car platforms have been widely developed by multiple companies and research groups with various sensorial configurations that are specifically suited for different environments and applications. A comprehensive survey of commercial and indigenous MMS consoles was presented by scholars who described the variety of sensorial specifications along with the mapping and classification of street furniture [18]. A popular commercial MMS product, Leica Pegasus is widely used to produce 3D coloured maps of urban regions by incorporating a high-tech rotating 2D laser scanner along with wide field of view (FOV) cameras and GNSS, as shown in Fig 2. The Teledyne Optech Lynx HS600 is another high-performance, survey-grade MMS product designed for capturing highly accurate 3D mapping data of urban environments and infrastructure. For detailed specifications, refer to the Leica Pegasus documentation [19] and the Teledyne Geospatial product page [20]. Many more similar MMS units have been produced in developed markets; however, they are notably costly and require high-end processing and computation facilities due to the high volume of data [21].

Researchers from renowned universities have developed economical MMS solutions using comparatively low-price 2D laser scanners and other sensors [22]. Some researchers at KAIST have introduced indigenous MMS prototypes using low-price laser scanners [23]. These systems are utilized for accurate urban mapping and road surface monitoring, as summarized in Table 1 and as shown in Fig 2. Another research group has presented an overview of MMS and associated sensors [24]. However, higher the range, accuracy and resolution of MMS, the cost becomes unbearable for numerous surveying tasks in developing markets.

**Table 1:** Summary of 2D mobile mapping systems.

Product	System Description	Applications
Leica Pegasus[19]	2x Lidar, 6x camera, GNSS/IMU Range:119m	Smart city mapping and surveying of road and on ground assets
MMS developed at University of Bonn [22]	2D Lidar, GNSS/IMU Range:119m	Development of map and structure modelling
MMS developed at KAIST [23]	3x 2D Lidar, 4x cameras, GPS/IMU, encoder, Range: 80m	3D urban mapping for roads and structural data

The MMS can generate 3D maps containing millions of Cartesian points that researchers have further filtered and segmented for the classification of road asset information [25]. Scholars have comprehensively reviewed various point cloud processing approaches for object detection and classification for large urban datasets [26]. The algorithms related to feature detection and segmentation have been discussed for different scenarios in different research works [27]. Many researchers have presented various classification techniques for static on-road and off-road objects [28,29]. Specifically, pole-like object detection has been carried out by scholars [30]. In contrast to these fascinating studies, this article presents an extraction method for electric poles and road edges using low-cost MMS-based moderately dense point cloud processing.

## 3. Designing of low-cost mobile mapping system

Based on the needs of the targeted market segments, first, the Computer Aided Designing (CAD) of reduced-priced MMS for car-based surveying applications was performed, and later, actual fabrication was carried out, as described in the following section.

### 3.1. Mechanical design of the car scanning system

**Fig 3:**
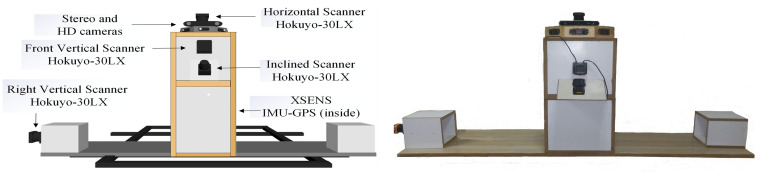
Custom-made car-based MMS (left) CAD model (right) Fabricated system.

The CAD of the MMS was designed in SolidWorks software, as shown in Fig 3 (left). Initially, an SS (stainless steel) mounting frame with dimensions of 75x24 cm^2^ was installed on the roof of the selected Suzuki Cultus car. A wooden sheet with dimensions of 80x40x10 cm^3^ was placed on the frame in which multiple wooden boxes were placed. A central box with dimensions of 40x40x60 cm^3^ is used to install the majority of the sensors, while two wooden cube boxes with dimensions of 20 cm^3^ are placed at the extreme right and left ends for possible improvements in sensor integration. The height of the central box is carefully selected to ensure that all the laser scanners can perceive the road and surrounding objects without hindering the roof of the car. The mounting platform was fabricated by using precision laser CNC cutters to minimize misalignments within 1mm range. The laser scanners have integrated on standard horizontal and vertical orientations. The first 2D Hokuyo-30LX scanner was mounted horizontally on the top plane of the central box to scan the XY plane of the surveying vicinity. The second Hokuyo-30LX scanner was installed vertically on the front plane of the central box to scan the YZ plane. The third Hokuyo-30LX scanner was installed at an inclination of 30° on the front plane to scan the environment with different angular views. A small octagonal wooden box with dimensions of 40x40x8 cm3 was placed on the top plane of the central box equipped with three HD Logitech cameras and one ZED stereo camera. The XSENS IMU is integrated in the center plane of the middle wood box, while the encoders are mounted on one rear wheel and other on the steering wheel of the car. One optional Hokuyo-30LX scanner was installed vertically in the right box. All the electronics, batteries and necessary cables were housed in the ground plane of the central box.

After completing the CAD model, the fabrication of the actual structure was carried out using thrifty manufacturing and all the components were installed as per the design, as shown in Fig 3 (right).

### 3.2. Instrumentation hardware design

**Fig 4:**
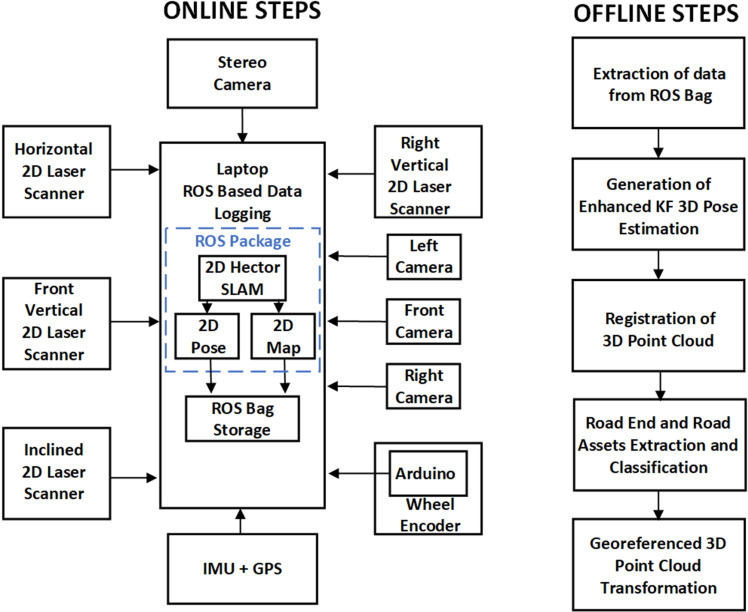
Instrumentation block diagram of MMS.

In order to interface all the sensorial components during surveying, a comprehensive instrumentation scheme was designed, as shown in the block diagram in Fig 4. All the sensorial information was processed and recorded at runtime. A 2D Hokuyo-30LX laser scanner has been selected as the primary source to perceive the surveying vicinity. The scanner has a range of 30 m with acceptable technical parameters, as observed in its detailed performance checking [31,32]. Multiple tests such as drift, range, color and material tests were performed to analyze the scanner in different environments for stability and repeatability from the respective datasheet. The scanned measured values were within the acceptable range and no significant deviations were observed. Three scanners were connected using a network switch to the Robot Operating System (ROS), which runs on a laptop. The ROS is an efficient open-source framework of various packages for acquiring, processing and recording live asynchronous and heterogeneous sensorial data streams. The horizontal laser scans are utilized to estimate the moving car 2D pose and its surrounding map by using the Hector SLAM package [33]. Both vertical and inclined laser scans were recorded to accumulate horizontal scans to generate a 3D map in the offline mode of the instrumentation scheme. An additional right vertical scanner was further interfaced with ROS laptop through a USB port. The XSENS MTi-G-710 IMU was selected and interfaced with the ROS to acquire the orientation, inertial and GPS data of the moving car. Two rotary encoders are also installed, with one located at the right back wheel of the car and the other coupled with the steering shaft, for collecting the vehicle’s rotational distance and heading throughout the journey [34]. Both encoders interfaced with the Arduino embedded board to serially transmit the live data to the ROS laptop. The IMU and encoder data feeds are utilized in offline mode to correct the pose of the moving vehicle. For live visual perception, three HD Logitech cameras interfaced with the ROS along with the ZED stereo camera.

## 4. Pose estimation and point cloud generation

This section explains the methodology for determining the pose of a moving vehicle and the development of a 3D point cloud map of the surveyed environment.

### 4.1. Kalman filter-based mobile platform localization

The localization of the moving vehicle is carried out to determine the six-degree-of-freedom (6-DOF) pose estimation in three-dimensional space, as represented by the state vector X described in equation 1.

$X={\left[x_{v}\;y_{v}\;z_{v}{\;\theta }_{vx}\;\theta_{vy\;}\theta_{vz}\right]}^{T}$ (1)

where (*x*_*v*_*,y*_*v,*_*z*_*v*_) and (*θ*_*vx*_*,θ*_*vy*_*,θ*_*vz*_*)* represent the pose components related to translational and rotational variations of the moving vehicle. The localization estimation is carried out in offline mode using MATLAB scripting by applying the KF technique to the recorded sensorial data feeds during the movement of the vehicle through IMU, wheel odometry, GPS and SLAM. Although online GPS and SLAM-based poses are available during vehicle motion, they are often too erroneous to mislead the actual localization of the vehicle. Therefore, the IMU-odometric data are further incorporated along with the GPS-SLAM values in the enhanced KF localization scheme. The IMU provides motion information of velocity and acceleration at 400 Hz in addition to odometric data; therefore, both are used in the prediction step of the KF implementation, while low-rate GPS-SLAM data are utilized in the correction phase. The linear velocity and acceleration data from IMU were modeled to determine the periodic translational changes using the standard linear motion equation. Let *∆ x*_*i*_ be the periodic x-axis translational change that can be modeled by using equation $\;{\Delta x}_{i}\;=\;v_{x_{i}}t+\frac{1}{2}\;{a_{x_{i}}t}^{2}$^,^ where the velocity $v_{x_{i}}\;$ and acceleration *a*_*xi*_ have sampled at time *t*. Similarly, the other translational changes in *∆y*_*i*_ and *∆z*_*i*_ are determined along with the ro*t*ational data feed from the IMU to represent the overall 3D localization state, as shown in equation 2.

$X_{I}={\left[{\Delta x}_{i}\;{\Delta y}_{i}\;{\Delta z}_{i}\;{\Delta \theta }_{xi}\;\Delta \theta_{yi\;}{\Delta \theta }_{zi}\right]}^{T}$ (2)

Similarly, the wheel odometric data provide translational measurements along with the steering shaft’s angular data to deliver the only 2D localization state at any time interval of the moving vehicle, as shown by equation 3. The IMU and odometric data are periodically utilized in the prediction phase of the KF.

$X_{O}={\left[{\Delta x}_{O}\;{\Delta y}_{O}\;0\;0\;0\;{\Delta \theta }_{O}\;\right]}^{T}$ (3)

The standard KF equations for the prediction phase to determine the state vector *X* and covariance matrix *P* are shown in equations 4 and 5 [35].

$X_{p}\;=\;AX_{n-1}\;+\;X_{u}$ (4)

$P_{p}=\;AP_{n-1}\;A^{T}+\;Q$ (5)

where, *X*_*P*_ and *P*_*P*_ are the predicted state vector and its associated covariance matrix, respectively. *X*_*u*_ is the current update provided by *X*_*I*_ or *X*_O_ based on their sampling rates. *A* is the unity system Jacobian matrix, while *Q* is the system noise matrix.

It is holding the known deviation values related to motion model uncertainties observed by the sensor’s responses during vehicle motion testing. In the correction phase of the KF, the GPS values are utilized to update the pose state vector. The XSENS IMU has built in GPS module to provide the localization information of the vehicle at a 4 Hz rate, as represented by equation 6.

$X_{G}={\left[{\Delta x}_{g}\;{\Delta y}_{g}\;0\;0\;0\;{\Delta \theta }_{g}\right]}^{T}$ (6)

Here, (*∆x*_*g*_*, ∆y*_*g*_) and *∆θ*_*g*_ indicate the position and orientation changes of the vehicle during each sampling time, respectively. Similarly, the 2D SLAM update *X*_*M*_ was generated at a 5 Hz rate to provide the position and orientation changes in each timestamp. Both *X*_*G*_ and *X*_*M*_ are used for KF corrective actions according to their update rates. However, due to GPS outages and SLAM errors due to the unavailability of sufficient distinct laser scan values, only acceptable outputs of both sources were utilized, which were validated by predefined thresholds and labeled *Z*_*n*_ in the KF formulation. The measurement update equation, $Y=Z_{n}-HX_{p}$, is applied for each distinct *X*_*G*_ or *X*_*M*_ to determine the residual between the current and predicted values. Here, *H* is the unity measurement Jacobian matrix. In continuation, the KF gain *K* is computed using $K=P_{p}H^{T}{\left(HP_{p}H^{T}+R\right)}^{-1}\;$,where *R* is the measurement noise matrix. Finally, the new estimates of *X* and *P* are computed using standard corrective equations, as shown in equations 7 and 8.

$X_{n}=\;X_{p}+KY$ (7)

$P_{n}=\;(I\;-\;KH)P_{p}$ (8)

**Fig 5:**
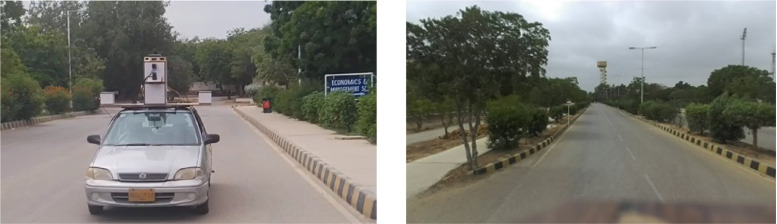
Campus Road traveling (left) Installed MMS on car (right) Road vicinity.

The presented pose estimation scheme was tested on a campus road by installing the developed MMS on a Suzuki Cultus car, as shown in Fig 5 (left). The car is driven inside the campus, and online sensorial data processing and logging are carried out on a high-end ROS-enabled laptop with a Core i911900H processor and intermediate level GPU (NVIDIA RTX 3060). The image captured from the front camera is shown in Fig 5 (right). This first field test vicinity is composed of roads, curbs, electric poles and vegetation.

**Fig 6:**
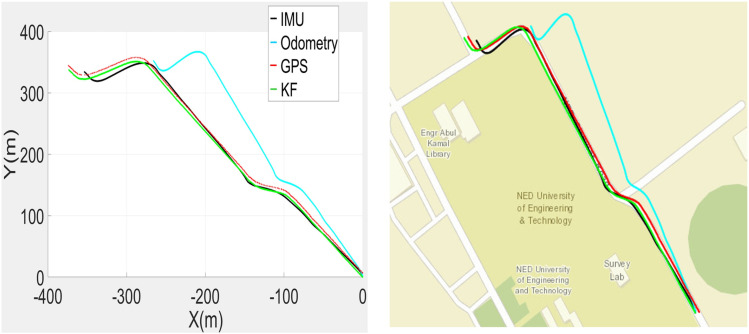
Plots of recorded and estimated poses (left) Poses in the SI unit (right) Poses on the georeferenced map.

The recorded SLAM, GPS, IMU and odometric data were used to estimate the poses of the car in the SI unit, as shown in Fig 6 (left). The wheel encoder poses have incrementally gained errors, while the IMU and GPS data feeds remained close to each other. The SLAM outputs were found to be too erroneous in the test due to the unavailability of sufficient perceived range values in the vicinity of the largely open space and were not employed in the correction phase. The estimated poses have transformed to lat-long values and are plotted in Fig 6 (right), where the traces of the estimated poses in green color have found almost overlapped with the actual road territory where the car was moved. Therefore, the estimated poses were further used for the development of 3D point clouds in the vicinity.

A quantitative analysis is conducted by comparing the actual and measured road edges of a straight road at multiple points. In order to validate the accuracy of extracted road edges, road boundary lane from original plot (Fig 6) is evaluated. It includes the average error and its root mean square error (RMSE) of the extracted road edge measured by particular sensors (GPS, IMU, wheel odometer) with the manually extracted edge. The error in each measurement of GPS, IMU, wheel odometer is listed in Table 2 along with proposed algorithm in this paper.

**Table 2:** Measurement of road edges extraction errors at a specific point.

Sensor/Method	Error (m)	RMSE (%)
GPS	0.045	4.5
IMU	0.085	8.5
Wheel odometer	0.175	17.5
Fusion method	0.032	3.2

### 4.2. Point cloud generation

The 3D point cloud map of the surveyed vicinity was developed in offline mode by MATLAB coding using the accumulation of all scans perceived by the integrated 2D laser scanners on the MMS. The horizontal scanner is selected as the reference scanner, and all other scans are transformed to its specified origin. The transformation of the vertical scans was performed using equation 9.

$P_{L21}=Trans(x_{2},y_{2},z_{2})Rot(Z,\theta_{z2})Rot(Y,\theta_{Y2})P_{L2}$ (9)

Here, *(x*_*2*_*,y*_*2,*_*z*_*2*_*)* and *(θ*_*y2*_*,θ*_*z2*_*)* represent the translational and rotational displacements of the vertical scanner w.r.t. the horizontal scanner, respectively. *P*_*L2*_ is the single scan point of the vertical scanner, which is transformed to *P*_*L21*_. All the vertical scan points were transformed and stored in a new scan vector, *P*_*L21C*_. Similarly, the inclined laser scans are transformed to a horizontal scanner using the standard transformation procedure shown in equation 10.

$P_{L31}=Trans(x_{3},y_{3},z_{3})Rot(Y,\theta_{Y3})P_{L3}$ (10)

Here, *(x*_*3*_*,y*_*3,*_*z*_*3*_*)* and *θ*_*y3*_ are the translational and rotational displacements w.r.t. the horizontal scanner. *P*_*L3*_ is the single scan point that is transformed to *P*_*L31*_, and all the points are transformed and stored in the *P*_*L31C*_ scan vector. The right vertical scans were transformed using the same procedure; however, they were not accumulated for point cloud mapping. Therefore, all the transformed scans were combined with the horizontal scan to develop a single referenced scan vector *T*_*LC,*_ as shown in equation 11. The scan $T_{LC}$ represents the 3D scan points perceived at a particular time stamp from the MMS and generally reaches approximately 3243 scan points.

$T_{LC}=P_{L1}+P_{L21C}+\;P_{L31C}$ (11)

Consequently, by using the estimated pose *(x*_*v*_*, y*_*v,*_ *z*_*v,*_ *θ*_*vz*_*)* of that particular timestamp, the scan *T*_*LC*_ can be transformed into world coordinates, as shown in equation 12. Due to testing on flat surfaces, the two angular pose parameters of the car (*θ*_*vx*_,*θ*_*vy*_) remained almost the same, so they were not incorporated into the transformation equation.

$T_{W}=Trans\left(x_{v},y_{v},z_{v}\right)Rot(Z,\theta_{vz})T_{LC}$ (12)

**Fig 7:**
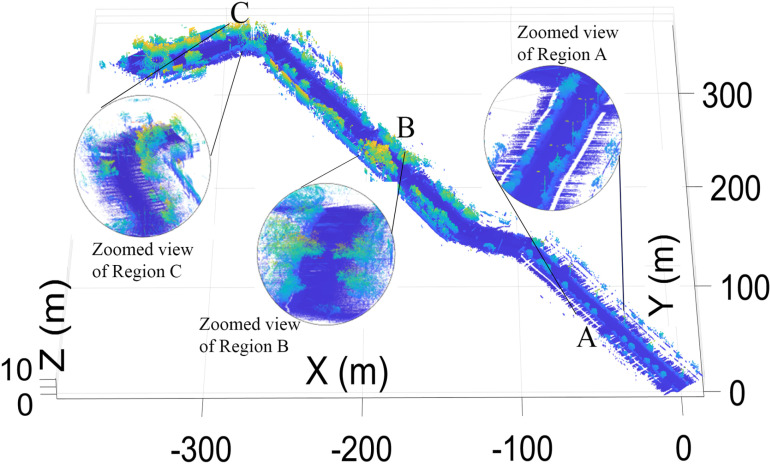
3D point cloud map representing the road surface, vegetation and electric poles.

Finally, by combining the individual transformed scans *T*_*W*_, the complete 3D point cloud map of the surveyed region was developed, as shown in Fig 7, which presents the road surface, curbs, vegetation and electric poles. The various sections of the map are enlarged and shown in the figure.

**Fig 8:**
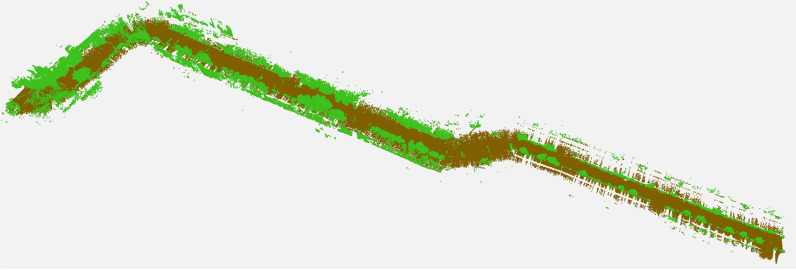
Classification of point clouds to separately represent ground and above ground entities.

The efficacy of the developed map was tested by uploading it on standard AUTODESK Recap software for improved visualization and processing, as shown in Fig 8. Point cloud classification has been applied to clearly represent the ground plane and road assets (vegetation, structures and objects) in distinct colors, as shown in the figure.

**Fig 9:**
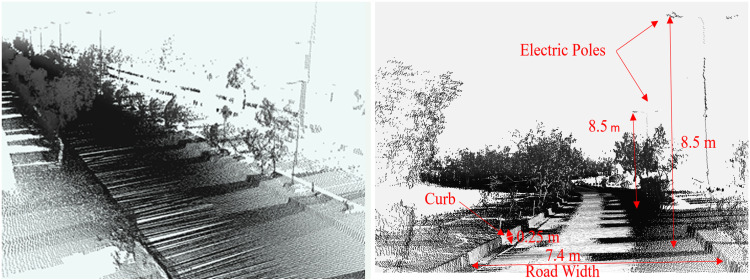
3D point cloud map visualization of the surveyed area (left) enlarged view and (right) road asset measurements.

In addition, any measurements related to road width, tree spacing or electric pole height can be easily measured on the software tool to provide information about the objects present in the surveyed vicinity, as shown in Fig 9.

**Fig 10:**
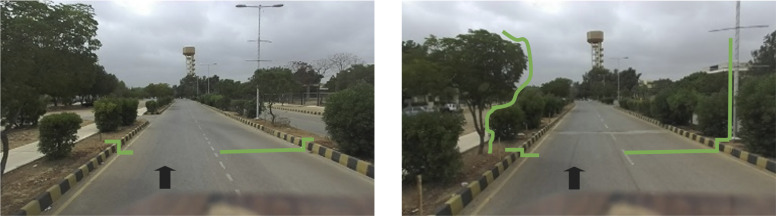
Vertical scanning views of the (left) road surface and (right) road assets.

The size of the generated 3D point cloud reaches thousands of cartesian points belonging to the surveyed region and encapsulates multiple objects and associated features. Therefore, various studies have classified point clouds to identify distinct objects [36]. However, the processing of overall point clouds is computationally expensive and requires expertise to reduce the classification burden [37]. Therefore, this research presents a manageable approach to process each vertical scan to identify the presence of roads and related assets such as electric poles or trees at any particular time stamp. The Hokuyo UTM-30LX is ideal among its league for applications such as pole detection, indoor/outdoor mapping, and small-scale surveying applications. The scanned measured values were within the acceptable range and no significant deviations were observed. The other scanning systems are significantly costly and need high-end computation systems for similar tasks. Different vertical scans of the road vicinities are shown in Fig 10, indicating that roads, electrical poles and trees appear during the movement of the car in the forward direction, as indicated by the black arrow.

**Fig 11:**
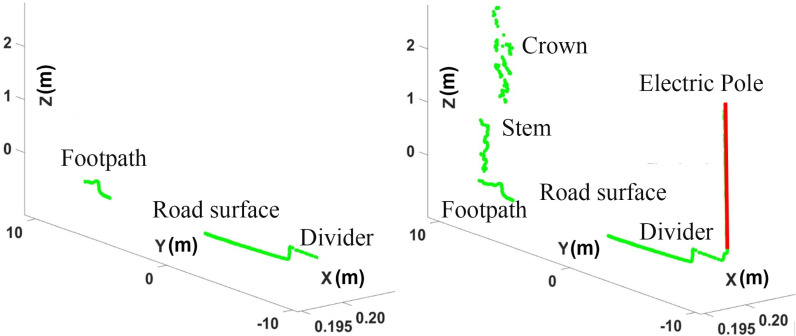
Multiple vertical scans of the (left) road surface and (right) various segments.

These scans were further plotted to indicate the variations in scan points belonging to different objects of the perceived region, as shown in Fig 11. Road surfaces, curbs and electric poles appear in distinct sets of linear points, while vegetation is reflected through a set of scattered points. Therefore, the segmentation of each vertical scan is performed using the Split and Merge algorithm to obtain linear and scattered sets of points in the scan [38]. All the horizontal linear sets of points are used to distinguish the road and curb segments along their endpoints. The vertical linear segment is used to identify the electric pole in the particular scan, as shown by the red color in Fig 11 (right).

**Fig 12:**
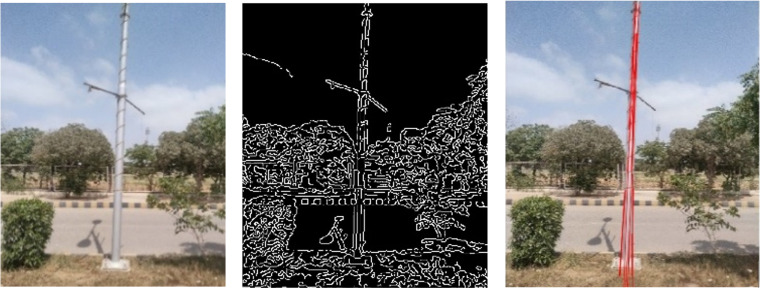
Vertical edge detection: (left) original image, (middle) extraction of edges and (right) vertical edges associated with the electric pole.

The validity of scan-based pole segmentation is tested using images from side cameras that were recorded during the same time stamps. Camera images are processed through multiple image processing tools, such as Canny edge detection and Hough transformation techniques [39]. Using these techniques, vertical line detection of electric poles has been carried out, as shown in Fig 12.

**Fig 13:**
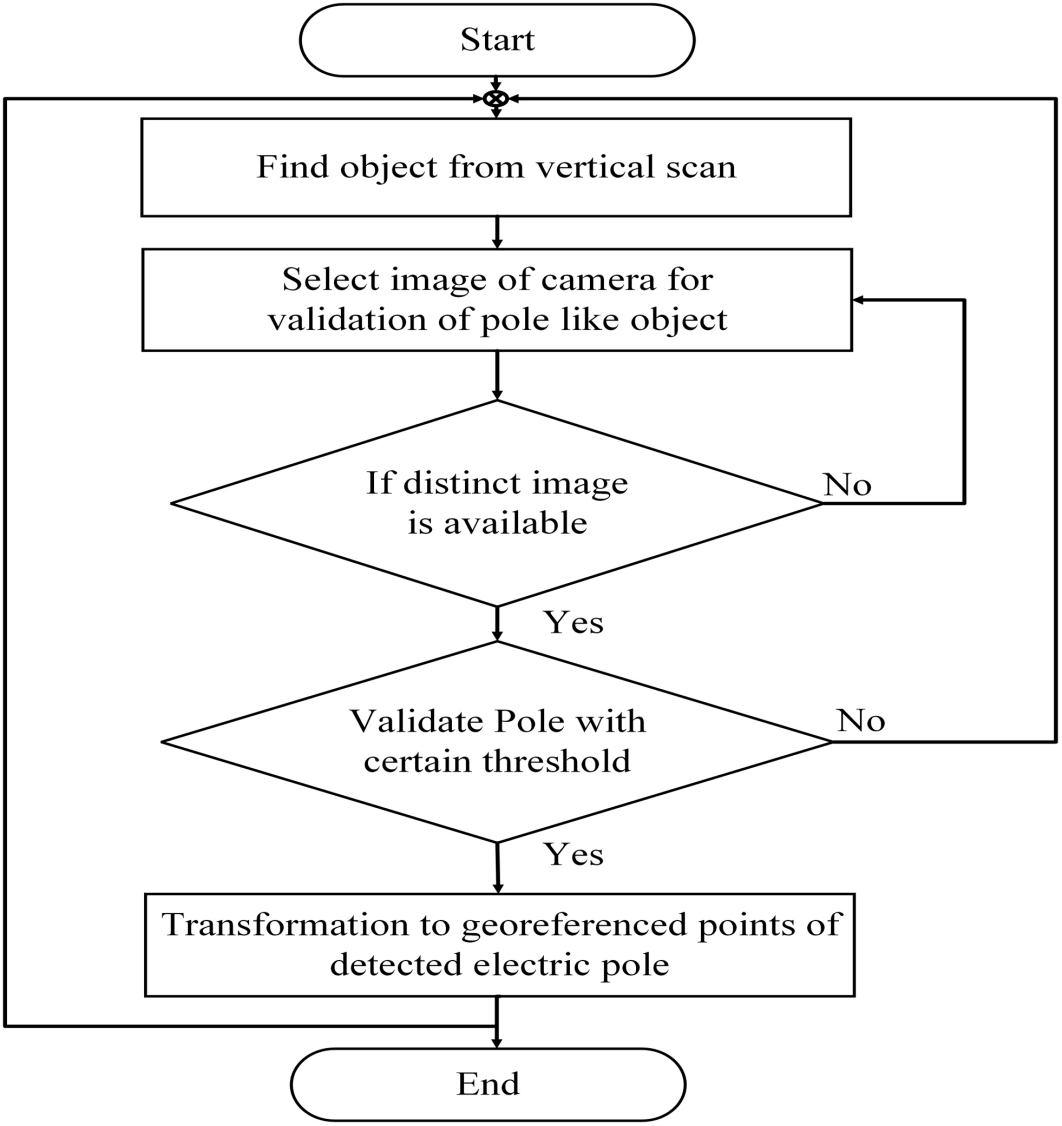
Workflow for classification of electric poles in the vicinity.

An offline workflow is established to automatically perform the segmentation and validation of electric poles, as shown in Fig 13. Each vertical scan is segmented during the 3D point cloud registration process to detect the electric pole, and simultaneously, the vertical edges of the corresponding image are detected. The edge detection scheme first converts the raw image into a gray image, and then the Canny edge detection filter is applied, followed by the Hough transformation to detect and mark the vertical object, as indicated by the red lines in the actual image [40]. The surveying time is greatly reduced for MMS as compared to traditional stationary or terrestrial scanners due to minimal surveying time of the same vicinity. This significantly reduces the setup time required for stationary scanners to change its location after each measurement. Moreover, from the developed MMS, street objects can be identified through only vertical scans during runtime which saves sufficient computational time for the classification of required objects as compared to conventional 3D point cloud processing for specific street objects [41]. This approach minimizes the computational demands, lowers the cost of high-end system requirements and enables faster processing which sums up to overall 60% less surveying and processing time.

**Fig 14:**
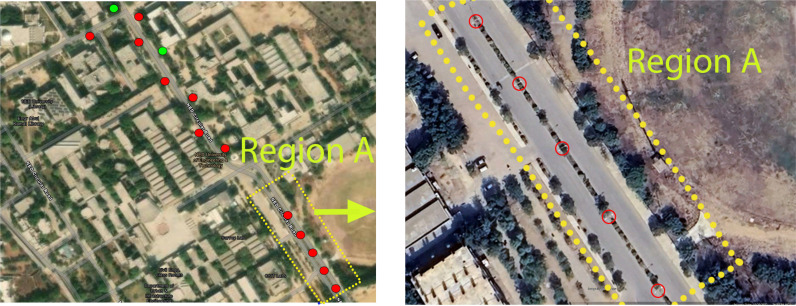
Extracted locations of the electric poles in region A (left) Complete view (right) Enlarged view.

The extracted road end points, curb boundaries and electric pole locations were transformed to georeferenced points and plotted on a standard global map, as shown in Fig 14. The overall 3D point cloud map was also transformed to geo-locations for viewing and analyzing the surveying results.

A comparison of the actual dimensions was performed with the measured dimensions of road width and pole height in Table 3. The gathered results are very nearly accurate to the ground truths. Moreover, the overall automatic detection rate of electric poles was 85% on the basis of manual visual inspection in the surveyed area. The undetected poles (marked with green) were missed due to the occlusion of trees in close vicinity, as shown in Fig 14.

**Table 3:** Measurement of road assets and electric pole detection.

	Measured (m)	Actual (m)	Error (%)	RMSE (m)
Road Width	7.46	7.5	0.46	0.035
Pole Height	8.55	8.6	0.53	0.047

## 5. Results

**Fig 15:**
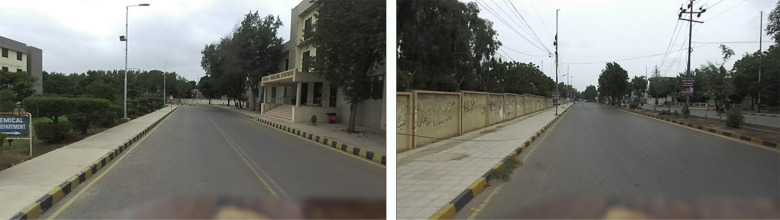
Front views of two surveyed regions (left) university campus road 2 and (right) main commercial road adjacent to the university.

The developed MMS was further tested in two separate urban regions, as shown in Fig 15. The first region is associated with the university road and is occupied by campus buildings, trees and electric installations.

**Fig 16:**
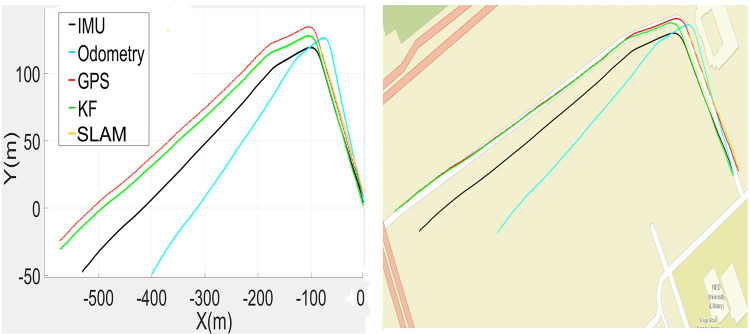
Plots of recorded and estimated poses (left) Poses in the SI unit (right) Poses on the georeferenced map.

**Fig 17:**
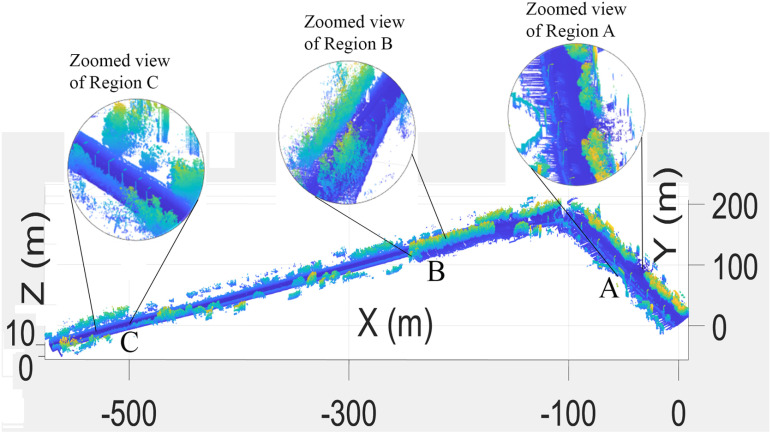
3D point cloud map representing the road surface, vegetation and electric poles.

The online MMS sensorial data recording was carried out during the movement of the car. The gathered GPS, IMU, SLAM and odometric localization results are utilized for enhanced vehicle pose estimations. All the results are plotted in Fig 16 (left). The odometric poses quickly started to deviate from the actual track, and later, the IMU and SLAM poses deteriorated. The GPS poses appeared closer to the traveled path. Based on these poses, the estimated KF-based poses were plotted and found to be quite accurate for 3D point cloud mapping. The estimated poses were transformed to georeferenced values for plotting on the standard global map and found to almost coincide with the campus road, as shown in Fig 16 (right). Using the estimated poses, the complete 3D point cloud map of the surveyed region was developed, as shown in Fig 17.

The map provides details of the road surface, curbs, nearby structures, vegetation and electric poles. The various sections of the map are enlarged and shown in the figure.

**Fig 18:**
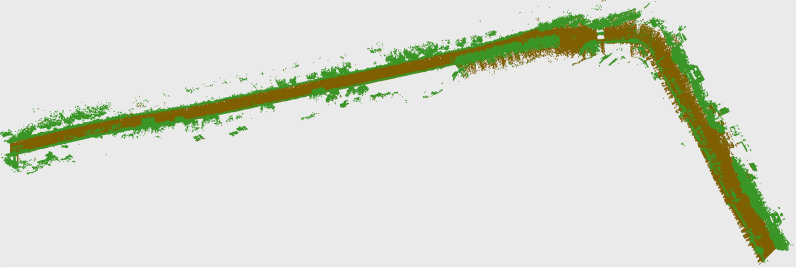
Classification of point clouds to separately represent ground and above ground entities.

The generated map was uploaded to the standard AUTODESK Recap software for improved visualization and processing, as shown in Fig 18. The point cloud classification was applied to view the ground plane and road assets, including vegetation, structures and objects in distinct colors, in this second field test, as shown in the figure.

**Fig 19:**
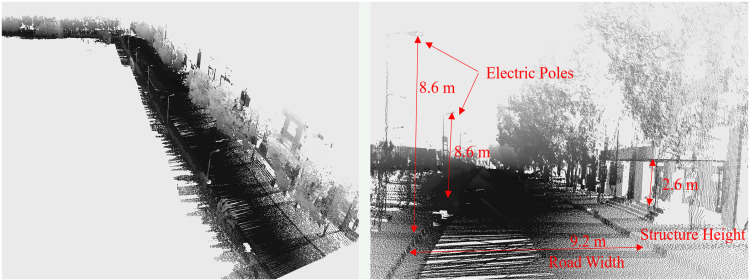
3D point cloud map visualization of the surveyed area (left) enlarged view and (right) road asset measurements.

To further analyze the generated map, measurements of the road surface and objects are performed and compared with the ground truth and found to be nearly accurate, as shown in Fig 19. However, due to the lateral movement of vehicles, few objects are partially scanned, which deteriorates their measurements.

**Fig 20:**
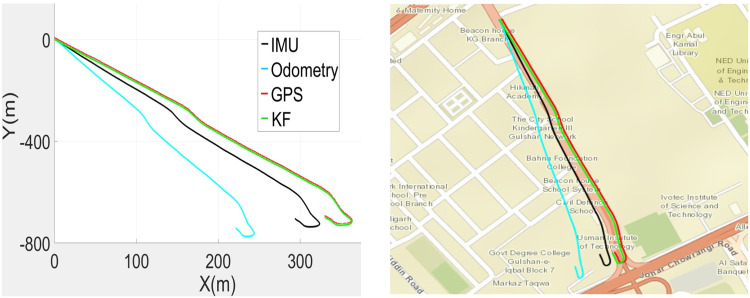
Plots of recorded and estimated poses (left) Poses in the SI unit (right) Poses on the georeferenced map.

To evaluate the comprehensive performance of the proposed localization scheme, a third field test was carried out on a commercial road adjacent to the campus. The traveled vicinity is composed of roads, curbs and multiple structures, including bridges, vegetation and electric poles, which have been recorded and analyzed. The recorded GPS, IMU and odometric data are used to estimate the poses of the car in the SI unit, as shown in Fig 20 (left), and its georeferenced values are shown in Fig 20 (right).

The wheel encoder repeated the trend to produce erroneous outputs, while the IMU measurements slightly deviated from the original path. The SLAM results were often found to be inaccurate due to the unavailability of distinct surrounding objects and were not used in estimation. Only the GPS data were close to the traveled trajectory. The car poses were estimated using all available data and are plotted in green, as shown in Fig 20. These poses were found to be almost overlaid with the real road territory where the car traveled. Consequently, the estimated poses were utilized for the development of the 3D point cloud of the surveyed region.

**Fig 21:**
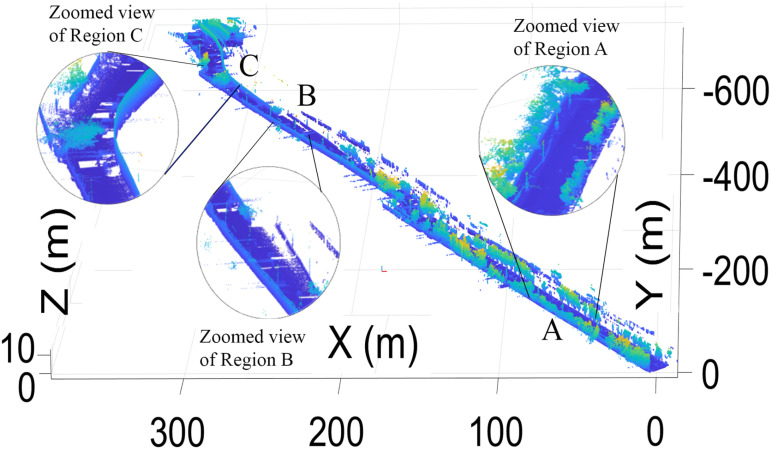
3D point cloud map representing the road surface, vegetation and electric poles.

The complete 3D point cloud map of the scanned territory is shown in Fig 21, which shows the road surface.

**Fig 22:**
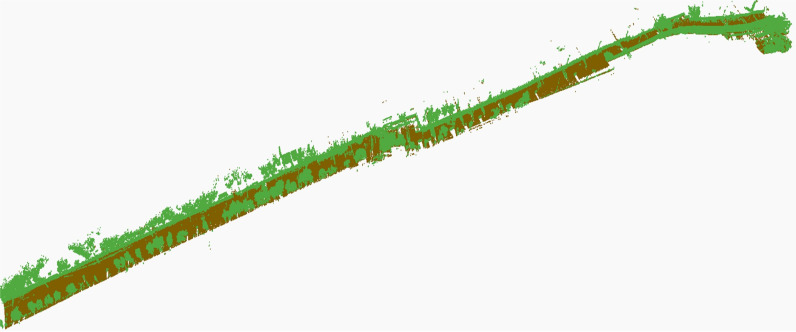
Classification of point clouds to separately represent ground and above ground entities.

The developed map was exported to Recap software for improved visual depiction and processing, as shown in Fig 22. Point cloud classification based on the z-gradient and shape has been applied to clearly represent the ground plane and road assets (vegetation, structures and objects) in distinct colours, as shown in the figure.

**Fig 23:**
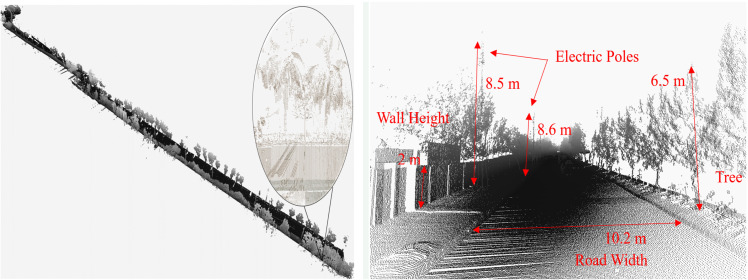
3D point cloud map visualization of the surveyed area (left) enlarged view and (right) road asset measurements.

Furthermore, measurements of road furniture, such as road width, tree spacing or electric pole height, can be easily measured with software to investigate the objects present in the surroundings, as shown in Fig 23.

The actual and measured dimensions of road width and pole height are summarized in Table 4. These measurements are found very close to the ground truth. Additionally, the detection algorithm extracted 19 poles out of 21 with a classification precision of 89% from visual inspection in the surveyed area. Some poles are not detected due to the lateral movement of vehicles during scanning and the presence of nearby trees adjacent to poles, as illustrated by the enlarged view in Fig 23 (left).

**Table 4:** Measurement of road assets and electric pole detection.

	Measured (m)	Actual (m)	Error (%)	RMSE (m)
Road Width	10.47	10.5	0.29	0.03
Pole Height	8.55	8.6	0.52	0.045

**Fig 24:**
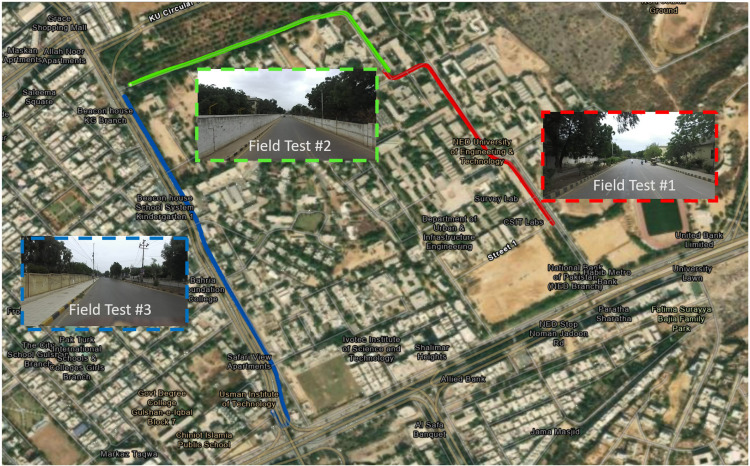
Localization plots of the three field tests in ArcGIS software.

Subsequently, differential GPS or highly accurate GNSS datasets were not available to validate the accuracy of localization; therefore, the results of the car localization were projected on ArcGIS Pro software, as shown in Fig 24 to trace the estimated paths on actual roads. All the localization results of the three discrete field tests are represented by red, green and blue colors for field tests 1, 2 and 3, respectively. The total distance traveled in all three tests reached to 5km long track. The estimated localization results are found to be satisfactory because they overlap with the actual road paths where the car has moved. The road scenes of each field test are shown within the enlarged windows inside Fig 24, which shows multiple trees, electric poles, structures and nearby objects.

**Fig 25:**
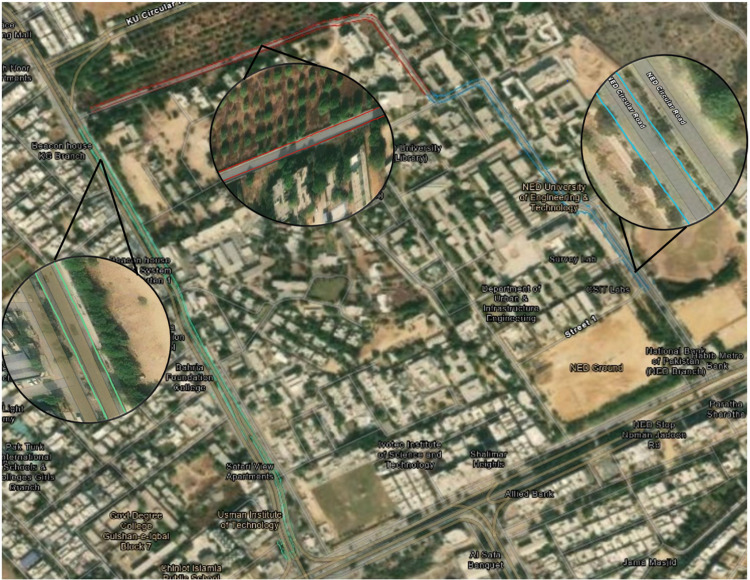
Plots of road ends in ArcGIS.

Furthermore, to evaluate the accuracy of the generated 3D map, the extracted road end points of all three field tests were exported to ArcGIS Pro software and visualized on satellite images for qualitative evaluation. As shown in Fig 25.

The measured road boundaries are well aligned with the corresponding satellite image and best fit with road ends of all three field tests, as shown in cyan, red and green colors. A few magnified views of the traveled regions are also shown. Therefore, the efficacy of the generated point cloud mapping mechanism has been successfully validated.

This road asset information is beneficial for digital map applications and road surveys evaluating the dimensions of roads, poles, structures and trees. This research work can provide 3D visualization and 2D segmentation information of road assets in optimal resources and requires less execution time due to the low density of point clouds and limited complexity as compared to other existing methods [42]. Moreover, our proposed method requires relatively less computation time for the segmentation of moderate point cloud as compared to presented work which use dense point clouds and 2D image qualifiers for the extraction of road scenes [28].

## 6. Conclusion

This research presents an indigenously developed economical MMS for low-range urban surveying applications. The operation of the system was tested on multiple roads to scan the surroundings during the movement of the MMS-installed car. The KF-based pose estimation of the car was carried out using all the MMS sensorial data, including encoder, IMU, GPS and 2D laser scan-based SLAM data. Using the estimated poses, the registration of all three distinct 2D laser scans have carried out to develop a 3D point cloud map of the surveyed vicinities. The point cloud map consists of a detailed Cartesian set of points belonging to road surfaces, buildings, vegetation and various other objects, such as electric poles. The efficacy of the map was further elaborated by uploading it in standard software such as Autodesk Recap to view and process the scanned data points. They have been successfully segmented to show ground and non-ground objects with accuracy. In addition, during the registration of individual scans, an automated workflow has been developed to classify the required road assets, such as electric poles and road curbs. The generated results achieved a good accuracy of 99% for indicating road ends and more than 85% of correct detection of electric poles in the presence of very high occlusion due to overlapping trees. Furthermore, all the developed 3D maps and extracted road ends, curbs and locations of electric poles were transformed to georeferenced maps to show the accuracy and usefulness of the generated results using the affordable MMS. Multiple views of the georeferenced maps are possible using standard tools such as ArcGIS. In addition, the overall surveying time is greatly reduced to 60% if compared with the existing surveying techniques in the targeted market due to less processing time required for single beam 2D laser scan. To further improve the localization accuracy and to colour the scanned points, stereo images are planned for use in future endeavours.

### Variables and constants

**Table:** 

$X,\;X_{I},\;X_{O},\;X_{G}$	State vector representation of car, measured by IMU, odometer and GPS	NA
$x_{v},\;y_{v},z_{v}$	Translational motion of car	m
$\theta_{vx}{,\theta }_{vy\;,}\theta_{vz}$	Rotational motion of car	rad
${\Delta x}_{i}\;{\Delta y}_{i}\;{\Delta z}_{i}\;$	Translational changes measured by IMU	m
${\Delta \theta }_{xi}\;\Delta \theta_{yi\;}{\Delta \theta }_{zi}\;$	Rotational changes measured by IMU	rad
${\Delta x}_{O}\;{\Delta y}_{O}$	Translational changes measured by odometer	m
${\Delta \theta }_{O}$	Rotational changes measured by odometer	rad
${\Delta x}_{g}\;{\Delta y}_{g}$	Translational changes provided by GPS	m
${\Delta \theta }_{g}$	Rotational changes provided by GPS	rad
$X_{p}\;,X_{n}$	Predicted mean value, current estimated mean value	NA
$P_{p},\;P_{n}$	Predicted variance value, current estimated variance value	NA
R, Q	Constant measurement variance, constant process variance	NA
$P_{L2},P_{L21},P_{L21C}$	Single scan, transformed scan w.r.t. horizontal scanner, combined scan of vertical scanner	NA
$P_{L3},P_{L31},P_{L31C}$	Single scan, transformed scan w.r.t. horizontal scanner, combined scan of inclined scanner	NA
$T_{C},T_{W}$	Transformed combined scan at particular timestamp, globally transformed scan	NA
RMSE	Root mean square error	m

## Supporting information

S1 Data (ZIP)

## Abbreviations

SLAM: Simultaneous Localization and Mapping
KF: Kalman Filter
ROS: Robot Operating System
MMS: Mobile Mapping System
GPS: Global Positioning System
IMU: Intertial Measurement Unit
UKF: Unscented Kalman Filter
GNSS: Global Navigation Satellite System

## References

[1] Chiang K, Tsai G, Zeng J. Mobile Mapping Technologies. In: The Urban Book Series. Springer; 2021. p. 439–465. doi: 10.1007/978-981-15-8983-6_25

[2] Elghazaly G, Frank R, Harvey S, Safko S. High-Definition Maps: Comprehensive Survey, Challenges, and Future Perspectives. IEEE Open J Intell Transp Syst. 2023;4:527–50. doi: 10.1109/ojits.2023.3295502

[3] Hassan M, Ullah M, Iqbal J. Towards autonomy in agriculture: Design and prototyping of a robotic vehicle with seed selector, Proceedings of the 2nd International Conference on Robotics and Artificial Intelligence (ICRAI);2016. pp. 37-44. IEEE; 2016. doi: 10.1109/icrai.2016.7791225

[4] Kukko A, Kaartinen H, Hyyppä J, Chen Y. Multiplatform Mobile Laser Scanning: Usability and Performance. Sensors. 2012;12(9):11712–33. doi: 10.3390/s120911712 PMC3478870 23112743

[5] Swaminathan HB, Sommer A, Becker A, Atzmueller M. Performance Evaluation of GNSS Position Augmentation Methods for Autonomous Vehicles in Urban Environments. Sensors (Basel). 2022;22(21):8419. doi: 10.3390/s22218419 36366117 PMC9657055

[6] Liu J, Guo G. Vehicle Localization During GPS Outages With Extended Kalman Filter and Deep Learning. IEEE Trans Instrum Meas. 2021;70:1–10. doi: 10.1109/tim.2021.309740133776080

[7] Mohamed SAS, Haghbayan M-H, Westerlund T, Heikkonen J, Tenhunen H, Plosila J. A Survey on Odometry for Autonomous Navigation Systems. IEEE Access. 2019;7:97466–86. doi: 10.1109/access.2019.2929133

[8] Agrawal P, Iqbal A, Russell B, Hazrati MK, Kashyap V, Akhbari F. PCE-SLAM: A real-time simultaneous localization and mapping using LiDAR data. In: IEEE Intelligent Vehicles Symposium (IV); 2017 Jun 11-14; Los Angeles, CA, USA. IEEE; 2017. p. 1752–1757. doi: 10.1109/ivs.2017.7995960

[9] Li X, Li S, Zhou Y, Shen Z, Wang X, Li X, et al. Continuous and Precise Positioning in Urban Environments by Tightly Coupled Integration of GNSS, INS and Vision. IEEE Robot Autom Lett. 2022;7(4):11458–65. doi: 10.1109/lra.2022.3201694

[10] Li S, Wang S, Zhou Y, Shen Z, Li X. Tightly Coupled Integration of GNSS, INS, and LiDAR for Vehicle Navigation in Urban Environments. IEEE Internet Things J. 2022;9(24):24721–35. doi: 10.1109/jiot.2022.3194544

[11] Lehtomaki M, Jaakkola A, Hyyppa J, Lampinen J, Kaartinen H, Kukko A, et al. Object Classification and Recognition From Mobile Laser Scanning Point Clouds in a Road Environment. IEEE Trans Geosci Remote Sensing. 2016;54(2):1226–39. doi: 10.1109/tgrs.2015.2476502

[12] Lu Y, Ma H, Smart E, Yu H. Real-Time Performance-Focused Localization Techniques for Autonomous Vehicle: A Review. IEEE Trans Intell Transport Syst. 2022;23(7):6082–100. doi: 10.1109/tits.2021.3077800

[13] Eldesoky A, Kamel AM, Elhabiby M, Elhennawy H. Real time localization solution for land vehicle application using low-cost integrated sensors with GPS. JART. 2020;18(4):214–28. doi: 10.22201/icat.24486736e.2020.18.4.1196

[14] Li X, Wang S, Li S, Zhou Y, Xia C, Shen Z. Enhancing RTK Performance in Urban Environments by Tightly Integrating INS and LiDAR. IEEE Trans Veh Technol. 2023;72(8):9845–56. doi: 10.1109/tvt.2023.3257874

[15] Liang Y, Muller S, Rolle D. Tightly Coupled Multimodal Sensor Data Fusion for Robust State Observation With Online Delay Estimation and Compensation. IEEE Sensors J. 2022;22(13):13480–96. doi: 10.1109/jsen.2022.3177365

[16] Wang Y, Lou Y, Song W, Tu Z, Wang Y, Zhang S. Simultaneous Localization of Rail Vehicles and Mapping of Surroundings With LiDAR-Inertial-GNSS Integration. IEEE Sensors J. 2022;22(14):14501–12. doi: 10.1109/jsen.2022.3181264

[17] Chang L, Niu X, Liu T. GNSS/IMU/ODO/LiDAR-SLAM integrated navigation system using IMU/ODO pre-integration. Sensors. 2020;20(17):4702. doi: 10.3390/s20174702 32825329 PMC7506683

[18] Wang Y, Chen Q, Zhu Q, Liu L, Li C, Zheng D. A Survey of Mobile Laser Scanning Applications and Key Techniques over Urban Areas. Remote Sensing. 2019;11(13):1540. doi: 10.3390/rs11131540

[19] Leica Geosystems. Leica Pegasus: Two Ultimate Mobile reality capture. Available from: https://pdf.directindustry.com/pdf/leica-geosystems/pegasus-two-ultimate-bro/14324-976365.html

[20] Teledyne Optech. Lynx HS600 | Teledyne Geospatial. Available from: https://www.teledyneoptech.com/en/products/mobile-survey/lynx-hs600

[21] Sairam N, Nagarajan S, Ornitz S. Development of Mobile Mapping System for 3D Road Asset Inventory. Sensors (Basel). 2016;16(3):367. doi: 10.3390/s16030367 26985897 PMC4813942

[22] Roh H, Jeong J, Cho Y, Kim A. Accurate Mobile Urban Mapping via Digital Map-Based SLAM. Sensors (Basel). 2016;16(8):1315. doi: 10.3390/s16081315 27548175 PMC5017480

[23] Heinz E, Holst C, Kuhlmann H, Klingbeil L. Design and Evaluation of a Permanently Installed Plane-Based Calibration Field for Mobile Laser Scanning Systems. Remote Sensing. 2020;12(3):555. doi: 10.3390/rs12030555

[24] Elhashash M, Albanwan H, Qin R. A Review of Mobile Mapping Systems: From Sensors to Applications. Sensors (Basel). 2022;22(11):4262. doi: 10.3390/s22114262 35684883 PMC9185250

[25] Pu S, Rutzinger M, Vosselman G, Oude Elberink S. Recognizing basic structures from mobile laser scanning data for road inventory studies. ISPRS Journal of Photogrammetry and Remote Sensing. 2011;66(6):S28–39. doi: 10.1016/j.isprsjprs.2011.08.006

[26] Che E, Jung J, Olsen MJ. Object Recognition, Segmentation, and Classification of Mobile Laser Scanning Point Clouds: A State of the Art Review. Sensors (Basel). 2019;19(4):810. doi: 10.3390/s19040810 30781508 PMC6412744

[27] Ma L, Li Y, Li J, Wang C, Wang R, Chapman MA. Mobile Laser Scanned Point-Clouds for Road Object Detection and Extraction: A Review. Remote Sensing. 2018;10(10):1531. doi: 10.3390/rs10101531

[28] Babahajiani P, Fan L, Kämäräinen J-K, Gabbouj M. Urban 3D segmentation and modelling from street view images and LiDAR point clouds. Machine Vision and Applications. 2017;28(7):679–94. doi: 10.1007/s00138-017-0845-3

[29] Zhao L, Yan L, Meng X. The Extraction of Street Curbs from Mobile Laser Scanning Data in Urban Areas. Remote Sensing. 2021;13(12):2407. doi: 10.3390/rs13122407

[30] Huang J, You S. Pole-like object detection and classification from urban point clouds. In: IEEE International Conference on Robotics and Automation (ICRA); 2015 May 26-30; Seattle, WA. IEEE; 2015. p. 3032-3038. doi: 10.1109/icra.2015.7139615

[31] Jafri SRUN, Shamim S, Faraz SM, Ahmed A, Yasir SM, Iqbal J. Characterization and calibration of multiple 2D laser scanners. PLoS One. 2022;17(7):e0272063. doi: 10.1371/journal.pone.0272063 35900977 PMC9333273

[32] Shamim S, Jafri SR un N. A Comparative Study of Multiple 2D Laser Scanners for Outdoor Measurements. INTERACT 2023. 2023:16. doi: 10.3390/engproc2023032016

[33] Kohlbrecher S, von Stryk O, Meyer J, Klingauf U. A flexible and scalable SLAM system with full 3D motion estimation. In: IEEE International Symposium on Safety, Security, and Rescue Robotics; 2011 Nov 1-3; Kyoto, Japan. IEEE; 2011. p. 155-160. doi: 10.1109/ssrr.2011.6106777

[34] Abdallah R, Wu B, Wan J. Real-Time Vehicle Localization Using Steering Wheel Angle in Urban Cities. In: IEEE International Conference on Mobility, Operations, Services and Technologies (MOST); 2023 Aug 20-23; Detroit, MI, USA. IEEE; 2023. p. 62-70. doi: 10.1109/most57249.2023.00015

[35] Urrea C, Agramonte R. Kalman Filter: Historical Overview and Review of Its Use in Robotics 60 Years after Its Creation. Journal of Sensors. 2021;2021(1):9674015. doi: 10.1155/2021/9674015

[36] Lehtomäki M, Jaakkola A, Hyyppä J, Kukko A, Kaartinen H. Detection of Vertical Pole-Like Objects in a Road Environment Using Vehicle-Based Laser Scanning Data. Remote Sensing. 2010;2(3):641–64. doi: 10.3390/rs2030641

[37] Guan H, Li J, Yu Y, Chapman M, Wang C. Automated Road Information Extraction From Mobile Laser Scanning Data. IEEE Transactions on Intelligent Transportation Systems. 2015;16(1):194–205. doi: 10.1109/tits.2014.2328589

[38] Borges G, Aldon M. A split-and-merge segmentation algorithm for line extraction in 2D range images. In: 15th International Conference on Pattern Recognition; 2000 Sep 3-7; Barcelona, Spain. IEEE; 2000. doi: 10.1109/icpr.2000.905371

[39] Hassanein A, Mohammad S, Sameer M, Ragab M. A Survey on Hough Transform, Theory, Techniques and Applications. arXiv. 2015. doi: 10.48550/arXiv.1502.02160

[40] Huang Q, Liu J. Practical limitations of lane detection algorithm based on Hough transform in challenging scenarios. International Journal of Advanced Robotic Systems. 2021;18(2). doi: 10.1177/17298814211008752

[41] Lo Y, Huang H, Ge S, Wang Z, Zhang C, Fan L. Comparison of 3D Reconstruction Methods: Image-Based and Laser-Scanning-Based. Proc of the 24th International Symposium on Advancement of Construction Management and Real Estate. Springer; 2021, pp. 1257-1266. doi: 10.1007/978-981-15-8892-1_88

[42] Yang B, Liu Y, Liang F, Dong Z. Using mobile laser scanning data for features extraction of high accuracy driving maps. Int Arch Photogramm Remote Sens Spatial Inf Sci. 2016;XLI-B3:433–9. doi: 10.5194/isprs-archives-xli-b3-433-2016

